# Knowledge, attitudes, and practices regarding self-medication among Generation Z university students*

**DOI:** 10.1590/1980-220X-REEUSP-2025-0573en

**Published:** 2026-06-22

**Authors:** Hemelly Nogueira Guimarães Silveira, Silvia Regina Secoli, Aline Carrilho Menezes, Mailson Marques de Sousa, Stéfanie de Souza Rocha Ferreira, Flávia de Oliveira, Silmara Nunes Andrade, Danilo Donizetti Trevisan

**Affiliations:** 1 Universidade Federal de São João del Rei, Programa de Pós-Graduação em Enfermagem, Divinópolis, MG, Brazil.; 2 Universidade de São Paulo, Escola de Enfermagem, Programa de Pós-Graduação em Enfermagem na Saúde do Adulto, São Paulo, SP, Brazil.; 3 Universidade Federal de São João del Rei, Departamento de Enfermagem, Divinópolis, MG, Brazil.; 4 Universidade Federal da Paraíba, Programa de Pós-Graduação em Enfermagem, João Pessoa, PB, Brazil.

**Keywords:** Health Knowledge, Attitudes, Practice, Self Medication, Students, Universities

## Abstract

**Objective:**

To assess the knowledge, attitudes, and practices of self-medication and factors associated with this behavior among students in the health field.

**Method:**

A cross-sectional study conducted at a public university in Minas Gerais with 237 students. Data collection was carried out using a structured form to obtain sociodemographic variables and information on knowledge, attitudes, and practices regarding self-medication. Descriptive analysis and regression modeling were performed.

**Results:**

The majority (84.8%) reported practicing self-medication and demonstrated a high level of favorability for the behavior; 57.7% demonstrated adequate knowledge. Students in the early grades were more likely to have inadequate knowledge. Favorable attitudes were significantly associated with the 18–20 age group and low income. The highest rates of self-medication were statistically linked to young people and those who self-identified as Black.

**Conclusion:**

The high incidence of self-medication and the false perception of technical autonomy highlight the medicalization of life as a response to academic pressures. The results demonstrated vulnerabilities associated with structural inequalities and reinforce the need for institutional interventions focused on medication safety.

## INTRODUCTION

In the context of health education, the model known as Knowledge, Attitude, and Practice (KAP) represents an essential tool. This systemic approach, which allows for the assessment of what people know, how they think, and how they act in the face of a given health problem or practice, is fundamental for identifying specific behavioral risks^(1)^. Knowledge guides attitudes, which in turn motivate practices, and consistent practices reinforce attitudes and expand upon specific knowledge^(1)^. KAP-type surveys are widely used to elucidate health behaviors, including self-medication, among university students^(2,3,4,5,6,7,8,9,10)^. Within this specific group, the consumption of medication without a prescription from a qualified professional is a recurring and highly prevalent practice, especially among students in the health field^(2,3,4,5,6,7,8,9,10,11,12)^.

Among university students, the motivations for self-medication are based on the search for time optimization and convenience in the face of the pressures of daily academic life. The immediate relief of common symptoms, such as headaches, flu-like symptoms, and dysmenorrhea, is the main justification for using medication without professional guidance^(4,5,6,7,8,9,10,11,12)^. In addition, gaps in the healthcare system, manifested in slow service and difficulty in scheduling appointments, act as determining factors that drive young people to this practice. However, this scenario favors the indiscriminate consumption of medications, increasing the risks of adverse events and posing a serious challenge to patient safety and rational use^(2,4,7,13)^. Self-medication, in addition to compromising the safe use of medications, generates unnecessary costs for the healthcare system resulting from delays in disease diagnosis, drug interactions, serious adverse reactions, and antimicrobial resistance^(10,13)^.

Research based on the KAP model demonstrates that factors such as educational level, income, gender, and cultural context are determining factors in the practice of self-medication^(5,10)^. In Brazil, however, studies applying this triad to university students in the health field are still in their early stages^(3)^. This is especially true regarding Generation Z. Individuals belonging to Generation Z, characterized by hyperconnectivity and a strong pursuit of autonomy, exhibit a vulnerability profile for making reckless health decisions^(14)^. Unrestricted access to digital information and the need for immediate problem-solving, when combined with academic pressures and daily stresses, increase the irrational consumption of over-the-counter medications^(15)^. In students in the health field, these behavioral traits synergistically converge with easy access to specialized literature and interaction with peers in the healthcare sector^(4,5,6,7,8,9,10,11)^.

In the academic context, although students have greater access to medications, especially in the practice of self-medication, and believe they have technical mastery, limited therapeutic training and underestimation of clinical risks, often masked by a false perception of technical autonomy resulting from early contact with healthcare, can lead to negligent behaviors^(4)^. This misguided view culminates in unsafe practices and the occurrence of adverse reactions^(2,9)^. In this scenario, the pursuit of immediate symptom relief constitutes a risky behavior that must be countered by active academic training^(4,5,6,7,8,9,10,11,12)^, focused on user safety and mitigation of the indiscriminate consumption of medications. Given this scenario, the intersection between KAP and self-medication among Generation Z university students emerges as a critical field of study and a challenge for educational institutions.

The university represents a strategic space for developing actions that discourage indiscriminate self-medication and promote the rational use of medicines, to train future professionals to recognize the limits of self-care and the importance of clinical monitoring. Therefore, this study aimed to evaluate the knowledge, attitudes, and practices of self-medication and factors associated with this behavior among health science students at a public university in Minas Gerais, with a view to supporting educational interventions and institutional policies that mitigate the risks associated with self-medication and strengthen the culture of safety and the safe use of medications.

## METHOD

### Design of Study

This is a cross-sectional study.

### Local

Study conducted at a federal public university located in the Central-West region of Minas Gerais.

### Selection Criteria

The study population consisted of students from the Nursing, Medicine, Pharmacy, and Biochemistry courses. The inclusion criteria were: age 18 or older and regular enrollment in the educational institution. Students without an active email address or who experienced technical difficulties receiving the research instrument via email were excluded.

### Sample Definition

The sample size calculation was based on a population of 692 students, considering the prevalence of self-medication of 63.9%, identified in a study with characteristics and objectives similar to the present work^(4)^. A margin of error of 5% and a confidence level of 95% were adopted, resulting in an estimated minimum sample size of 237 participants. To ensure representativeness, the sample was stratified proportionally by course and academic period.

### Data Collection

The collection was carried out online, between February and August 2024, through a form from *Google Forms,* sent via institutional e-mail with the invitation and access link. After accepting the Free Informed Consent Form, participants answered a validated questionnaire^(3)^ entitled “Knowledge, attitudes, and practices of self-medication among university students”, consisting of 38 items divided into three sections:

*Section A* – Sociodemographic, academic, and health characterization: comprised of questions aimed at identifying age, sex, race/color, family income, current housing, type of educational institution, course and semester, access to health services, and assessment of health status.

*Section B* – Knowledge and practice regarding self-medication: obtained indirectly from responses about the practice of self-medication. To assess knowledge, information from the Drugs.com database (open access) was used as a reference^(16)^. Knowledge was considered adequate if the information reported by the participants was deemed correct by the database and if they did not fill any field of doubts regarding the indication, contraindication, dosage/administration, maximum duration of use, potential adverse reactions, drug interactions, precautions, or other questions.

*Section C* – Beliefs and attitudes about self-medication: this was measured using a five-point Likert-type scale ranging from one (strongly disagree) to five (strongly agree). The sum of the answers divided by the number of items generates a score that can range from 1 to 5; the higher the score, the more likely the student is to practice self-medication.

The practice of self-medication was analyzed based on reports of medication use in the last 15 days, including symptoms, type and use of medications (name, dose, route, dosage, perceived effects and justifications).

### Data Analysis and Treatment

Statistical analysis was performed in the software *Statistical Package for the Social Sciences* (SPSS), version 21.0. Descriptive statistics were used, with absolute and relative frequencies for categorical variables, and means with standard deviations (SD) or medians with interquartile ranges (IQR), depending on the data distribution. The normality of the data was assessed using the Kolmogorov-Smirnov test. For the associations, Pearson’s chi-square test was used. The dependent variables were: self-medication practice (dichotomous), knowledge (adequate/inadequate), and attitude (≤ 3 or >3). The independent variables encompassed the demographic, socioeconomic, academic, and medication use blocks. Variables with p < 0.20 in the bivariate analysis were included in the logistic regression model for calculating the Odds Ratio (OR). A significance level of p < 0.05 was adopted.

### Ethical Aspects

The study was approved by the institution’s Research Ethics Committee (Opinion No. 4.055.445, CAAE 30696620. 0.0000.5545) and followed all recommendations of Resolution No. 466/2012 of the National Health Council (CNS). All participants signed the FICF digitally, guaranteeing anonymity and the exclusive use of their data for scientific purposes.

## RESULTS

Of the 252 questionnaires received, 15 were excluded because they were incomplete. Therefore, the sample consisted of 237 respondents. Table 1 illustrates the total number of students evaluated, and the statistically significant differences occurred in the following variables: year of study (p < 0.001) in relation to knowledge; age group (p = 0.046) and income (p < 0.010) in relation to attitude; and age group (p = 0.028), sex (p = 0.048) and skin color (p = 0.001) in relation to the practice of self-medication.

**Table 1:** Sociodemographic, academic, and health characteristics in relation to the knowledge, attitudes, and practices of university students – Divinópolis, MG, Brazil, 2024.

Student characteristics	Knowledge (n = 201)				Attitudes (n = 237)				Practices (n = 237)		
	Adequate n (%)	Inadequate n (%)	p**		≤ 3.0 n (%)	> 3.0 n (%)	p**		Yes n (%)	No n (%)	p**
Age range											
≥ 18 and ≤ 20 years old	22 (10.9)	28 (13.9)	0.066		11 (4.6)	54 (22.8)	**0.046**		50 (21.1)	15 (6.3)	**0.028**
> 20 and ≤ 23 years old	50 (24.9)	33 (16.4)			32 (13.5)	67 (28.3)			83 (35.0)	16 (6.8)	
> 23 and ≤ 25 years old	44 (21.9)	24 (11.9)			25 (10.5)	48 (20.3)			68 (28.7)	5 (2.1)	
Sex											
Female	100 (49.8)	76 (37.8)	0.324		56 (23.6)	147 (62.0)	0.234		176 (74.3)	27 (11.4)	**0.048**
Male	16 (8.0)	09 (4.5)			12 (5.1)	22 (9.3)			25 (10.5)	9 (3.8)	
Undergraduate course											
Nursing	32 (15.9)	25 (12.4)	0.822		26 (11.0)	46 (19.4)	0.225		57 (24.1)	15 (6.3)	0.232
Medicine	25 (12.4)	15 (7.5)			10 (4.2)	33 (13.9)			40 (16.9)	3 (1.3)	
Pharmacy	34 (16.9)	23 (11.4)			22 (9.3)	46 (19.4)			57 (24.1)	11 (4.6)	
Biochemistry	25 (2.4)	22 (10.9)			10 (4.2)	44 (18.6)			47 (19.8)	7 (3.0)	
Year of study											
First and Second Year	38 (18.9)	60 (29.9)	**0.001**		32 (13.5)	90 (38.0)	0.688		98 (41.4)	24 (10.1)	0.245
Third and Fourth Year	59 (29.4)	22 (10.9)			29 (12.2)	64 (27.0)			81 (34.2)	12 (5.1)	
Fifth and Sixth year	19 (9.5)	03 (1.5)			07 (3.0)	15 (6.3)			22 (9.3)	0 (0.0)	
Monthly family income*											
≤ 01 MW	24 (11.9)	12 (6.0)	0.675		08 (3.4)	37 (15.6)	**0.010**		36 (15.2)	9 (3.8)	0.497
> 01 MW and ≤ 02	23 (11.4)	19 (9.5)			14 (5.9)	35 (14.8)			42 (17.7)	7 (3.0)	
> 02 MW and ≤ 03	24 (11.9)	20 (10.0)			13 (5.5)	41 (17.3)			44 (18.6)	10 (4.2)	
> 03 MW	45 (22.4)	34 (16.9)			33 (13.9)	56 (23.6)			79 (33.3)	10 (4.2)	
Race/Color											
White	72 (35.8)	53 (26.4)	0.865		41 (17.3)	103 (43.5)	0.885		125 (52.7)	19 (8.0)	**0.001**
Brown	34 (16.9)	23 (11.4)			17 (7.2)	45 (19.0)			57 (24.1)	05 (2.1)	
Black	10 (5.0)	09 (4.5)			10 (4.2)	21 (8.9)			19 (8.0)	12 (5.1)	
Access to the Health Service											
Exclusive audience	60 (29.9)	44 (21.9)	0.415		31 (13.1)	90 (38.0)	0.296		104 (43.9)	17 (7.2)	0.613
Public/Health Plan	46 (22.9)	29 (14.4)			42 (17.7)	46 (19.4)			75 (31.6)	13 (5.5)	
Health Plan/Private	10 (5.0)	12 (6.0)			05 (2.1)	23 (9.7)			22 (9.3)	6 (2.5)	
Health status classification											
Very good	26 (12.9)	15 (7.5)	0.098		12 (5.1)	38 (16.0)	0.241		41 (17.3)	9 (3.8)	0.617
Good	59 (29.4)	35 (17.4)			38 (16.0)	74 (31.2)			94 (39.7)	18 (7.6)	
Bad	31 (15.4)	35 (17.4)			18 (7.6)	57 (24.1)			66 (27.8)	9 (3.8)	

*Minimum wage in 2024: R$1412.00;

**Chi-Square Test.

Most respondents (84.8%) reported self-medicating, and more than half (57.7%) reported this practice three or more times in the last 15 days. Regarding consumption, almost the entire sample (96.5%) used over-the-counter medications, with analgesics being the most common (70.1%), and no adverse reactions were reported (88.5%). The main health reasons included headache (88.6%) and respiratory diseases (67.2%). Slightly more than half (57.7%) of the sample demonstrated adequate knowledge about the medications used, and the majority (76.6%) stated that personal knowledge was the main source of information for the practice of self-medication (Table 2).

**Table 2:** Practices and knowledge of university students regarding self-medication – Divinópolis, MG, Brazil, 2024.

Knowledge and practices of self-medication	n (%)
Practice of self-medication	201 (84.8)
Reason for self-medication*	
Headache	178 (88.6)
Upper airway problems	135 (67.2)
Menstrual cramps	84 (41.8)
Musculoskeletal pain	76 (37.8)
Gastrointestinal problems	72 (35.8)
Emotional problems	65 (32.3)
Contraception	56 (27.9)
Sleep problems	52 (25.9)
Fever	52 (25.9)
Skin problems	50 (24.9)
Justification for use*	
Personal decision based on prior knowledge	154 (76.6)
Repetition by personal decision due to prior prescription	85 (42.3)
Pharmacist’s recommendation	30 (14.9)
Family member recommendation	24 (11.9)
Recommendation from friends	16 (8.0)
Clerk’s Recommendation	7 (3.5)
Others	4 (2.0)
Knowledge*	
Adequate	116 (57.7)
Inadequate	85 (42.3)
Causes of inadequate knowledge (n = 85)	
Doubts about usage time	42 (49.4)
Questions about adverse reactions	34 (40.0)
Doubts about dosage	30 (35.3)
Doubts about drug interactions	27 (31.8)
Doubts about contraindications	25 (29.4)
Doubts about precautions during use	21 (24.7)
Doubts about referrals	6 (7.0)
Dosage error during use	5 (5.9)

*In the variables 'Reason for self-medication', 'Justification for use' and 'Knowledge', the percentages were calculated based on the total number of students who reported practicing self-medication (n = 201). The sum of the absolute numbers exceeds the total number of participants due to the possibility of multiple responses.

Table 3 presents the responses regarding attitudes towards self-medication. The median attitude toward self-medication was 3.41 (QT25 2.932 - QT75 3.864). The issues showing the highest attitude scores, that is, the greatest favorability for carrying out the behavior, involved the influence of the ease of buying over-the-counter medications (77.6%), the use of medications to solve common problems (75.9%), the practice of self-medication for quick relief and convenience (75.5%), and the waiting time for an appointment with a health professional (70.0%).

**Table 3:** Distribution of responses to items on the questionnaire about university students' attitudes towards self-medication – Divinópolis, MG, Brazil, 2024.

Attitudes towards self-medication	I totally disagree n (%)	I disagree n (%)	I don't know n (%)	I agree n (%)	Totally agree n (%)
1. Difficulties accessing the healthcare system and the decision to self-medicate.	40 (16.9)	26 (11.0)	55 (23.2)	52 (21.9)	64 (27.0)
2. Use of medication for common problems.	15 (6.3)	22 (9.3)	20 (8.4)	53 (22.4)	127 (53.6)
3. Use of medication in emergencies.	20 (8.4)	26 (11.0)	35 (14.8)	67 (28.3)	89 (37.6)
4. Use of medications recommended by family or friends.	54 (22.8)	51 (21.5)	65 (27.4)	36 (15.2)	31 (13.1)
5. Waiting time for an appointment with a healthcare professional and the decision to self-medicate.	23 (9.7)	22 (9.3)	26 (11.0)	49 (20.7)	117 (49.4)
6. Lack of trust in guidance from a healthcare professional and the decision to self-medicate.	73 (30.8)	45 (19.0)	33 (13.9)	41 (17.3)	45 (19.0)
7. Reusing old prescriptions and the ease of self-medication.	49 (20.7)	32 (13.5)	44 (18.6)	52 (21.9)	60 (25.3)
8. Having a stock of medications.	31 (13.1)	32 (13.5)	31 (13.1)	43 (18.1)	100 (42.2)
9. The influence of drug advertisements on the decision to self-medicate.	131 (55.3)	29 (12.2)	27 (11.4)	26 (11.0)	24 (10.1)
10. Seeking information for self-medication.	29 (12.2)	22 (9.3)	40 (16.9)	52 (21.9)	94 (39.7)
11. Lack of time to seek a healthcare professional and the decision to self-medicate.	21 (8.9)	37 (15.6)	31 (13.1)	58 (24.5)	90 (38.0)
12. Influence of previously used medications and the decision to self-medicate.	14 (5.9)	25 (10.5)	49 (20.7)	61 (25.7)	88 (37.1)
13. Family influence on self-medication.	46 (19.4)	23 (9.7)	43 (18.1)	55 (23.2)	70 (29.5)
14. The practice of self-medicating to reduce absences from school or work.	46 (19.4)	18 (7.6)	45 (19.0)	42 (17.7)	86 (36.3)
15. The relationship between self-medication and maintaining an active role in healthcare.	55 (23.2)	42 (17.7)	64 (27.0)	49 (20.7)	27 (11.4)
16. The relationship between reducing costs associated with consultations and examinations and self-medication.	44 (18.6)	48 (20.3)	51 (21.5)	38 (16.0)	56 (23.6)
17. Decision regarding the practice of self-medication involving minor health problems.	56 (23.6)	38 (16.0)	43 (18.1)	51 (21.5)	49 (20.7)
18. Safety for the practice of self-medication.	18 (7.6)	49 (20.7)	77 (32.5)	61 (25.7)	32 (13.5)
19. The influence of the ease of purchasing over-the-counter medications and the practice of self-medication.	12 (5.1)	14 (5.9)	27 (11.4)	54 (22.8)	130 (54.9)
20. The practice of self-medication for quick relief and convenience.	12 (5.1)	21 (8.9)	25 (10.5)	68 (28.7)	111 (46.8)
21. Agreement that over-the-counter medications may be accessible for self-medication.	52 (21.9)	41 (17.3)	25 (10.5)	47 (19.8)	73 (30.4)
22. Friendship with healthcare professionals or students and its influence on self-medication.	33 (13.9)	30 (12.7)	30 (12.7)	59 (24.9)	85 (35.9)

In the logistic regression analysis, it was identified that first-year students (OR: 10.00; 95% CI: 2.77–35.10) had a higher chance of having inadequate knowledge; those who rated their health status as good (OR: 0.525; 95% CI: 0.27–0.99) had a lower chance of having inadequate knowledge. Students aged 18–20 years (OR: 1.35; 95% CI: 1.32–7.75) with a family income ≤ one minimum wage (OR: 2.72; 95% CI: 1.13–6.55) were significantly more likely to have a more favorable attitude toward self-medication behavior compared to other age groups and income levels. The variables age between 18 and 20 years (OR: 4.46; 95% CI: 1.43–13.90) and black race/color (OR: 4.15; 95% CI: 1.74–9.91) were associated with a greater chance of self-medication; conversely, being male was related to a lower chance of this practice (OR: 0.36; 95% CI: 0.14–0.94).

## DISCUSSION

The results of this study revealed a high rate of self-medication among university students in the health sciences. Most participants reported self-medication, with a frequency of more than three times in over half of the cases (57.7%). This behavior was primarily motivated by the search for immediate solutions to relieve symptoms related to headaches and the consumption of analgesics.

Regarding knowledge, although more than half of the students (57.7%) demonstrated adequate understanding of self-medication, this rate is lower than in previous studies and insufficient to guarantee medication safety^(4,7,10)^. The lack of awareness of risks among incoming students reinforces the idea that including content on pharmacology and the rational use of medicines in health science curricula should not aim “conscious self-medication,” but rather train students to recognize the need for professional help and to combat irrational use^(13,17)^. Education should focus on preventing harm and deconstructing the idea that technical knowledge authorizes self-prescription.

Favorable attitudes towards self-medication reflect traits of Generation Z, such as the search for autonomy and practicality. However, this autonomy manifests itself as a digital vulnerability, where the ease of access to information and over-the-counter medications outweighs clinical caution. This generational need for immediacy, coupled with digital behavior, especially the frequent recourse to the Internet and social media, masks the inherent risks of the practice^(15,18)^. This deficit in risk perception requires educational institutions to examine the impact of the “online self-diagnosis” culture.

The reuse of old prescriptions (42.3%) and self-confidence based on previous experiences reveal a failure in the perception of safety. Barriers to accessing healthcare services, such as long waiting times, staff shortages, and high costs, while real and identified by other authors^(9,13,18)^, do not justify self-medication; rather, they represent structural problems that can lead individuals to adopt risky practices, potentially compromising the user’s long-term safety. Socioeconomic and racial factors influenced self-medication behavior, with a higher prevalence among students from lower-income families and those who self-identified as Black. These findings suggest that self-medication may reflect inequalities in access^(6)^. However, they also signal the medicalization of life as a palliative response to systemic and social inequities, including experiences of discrimination, distrust in the health system, and the influence of popular knowledge^(19)^.

The significant consumption of analgesics, which were used to relieve headaches, a prevalent complaint consistent with other authors^(10,12)^, suggests a potentially critical intersection between self-medication and the phenomenon of the medicalization of life in the university context. This environment often pushes the student towards a performance-driven logic that results in psychological suffering, for which the only treatment tends to be medication^(20)^. Thus, it is plausible to infer that, under high levels of stress and anxiety, Generation Z students resort to analgesics for the immediate relief of somatic manifestations (such as tension headaches) resulting from emotional pressures^(21)^. Consequently, the use of analgesics to mitigate psychological distress disguised as physical pain constitutes a risky behavior that masks emotional distress and reinforces the search for immediate pharmacological solutions, to the detriment of comprehensive care strategies and the rational use of medications.

Dependence on the Internet as a source of information (*YouTube*, *Instagram*, *TikTok*) potentiate the spread of information lacking scientific basis^(22,23)^. The fact that isolated technical knowledge does not prevent misuse, a finding evidenced among most participants in the different courses, indicates that academic training (at different levels) needs to evolve from a passive transmission of information to the construction of critical attitudes that discourage the use of medication without professional supervision. Understanding this behavior is essential for effective interventions.

However, what is often labeled as the “autonomy” of Generation Z actually reinforces the medicalization of life and indiscriminate use, to the detriment of a collective and safe vision of health^(21)^. This individualistic stance is concerning in healthcare training, as it can compromise future ethical practice and the ability to provide humane and technically responsible care. Therefore, educational institutions should invest in educational campaigns to fight self-medication and promote the rational use of medicines. These actions should utilize digital strategies to offer real care alternatives, such as promoting telemedicine and counseling with qualified professionals, dissociating the practice of self-medication from the concept of self-care^(8,21,23)^. Online community engagement and real-life stories can help change behavior, focusing on safety and clinical responsibility^(24)^.

Despite the limitations of recall bias resulting from self-reported data collection and the restricted sample size, the findings are crucial because they focus on the new generation of healthcare professionals. It is essential that health science curricula incorporate critical professional practice, focused on mitigating drug consumption and strengthening regulatory policies. The adoption of the KAP model should therefore serve as a basis for multidisciplinary strategies that protect students and the healthcare system from the irrational use of medication.

## CONCLUSION

The gap in technical knowledge identified in a significant portion of the students highlights the vulnerability of this group to health risks and reinforces the phenomenon of the medicalization of life as a response to academic and social demands. Furthermore, the disparities observed among lower-income students and those who self-identify as Black highlight the impact of structural inequalities on access to quality health information. It is concluded that there is an imperative need to implement educational strategies in higher education that transcend pharmacological theory, focusing on combating the irrational use of drugs and promoting medication safety, aiming to train critical professionals committed to safe, ethical, and equitable care.

## Data Availability

All the data supporting the results of this study were published in the article itself.
