# Supplementary material for: Knowledge, attitudes, and practices regarding self-medication among Generation Z university students
Source: Rev Esc Enferm USP. 2026 Jun 22;60:e20250573. doi: 10.1590/1980-220X-REEUSP-2025-0573en (PMC13286582; doi:10.1590/1980-220X-REEUSP-2025-0573en)
Supplement: Translation

# Translation: Conhecimento, atitudes e práticas sobre a automedicação em estudantes universitários da geração Z*

**DOI:** 10.1590/1980-220X-REEUSP-2025-0573pt

**Authors:** Hemelly Nogueira Guimarães Silveira, Silvia Regina Secoli, Aline Carrilho Menezes, Mailson Marques de Sousa, Stéfanie de Souza Rocha Ferreira, Flávia de Oliveira, Silmara Nunes Andrade, Danilo Donizetti Trevisan

**Affiliations:** 1 Universidade Federal de São João del Rei, Programa de Pós-Graduação em Enfermagem, Divinópolis, MG, Brasil.; 2 Universidade de São Paulo, Escola de Enfermagem, Programa de Pós-Graduação em Enfermagem na Saúde do Adulto, São Paulo, SP, Brasil.; 3 Universidade Federal de São João del Rei, Departamento de Enfermagem, Divinópolis, MG, Brasil.; 4 Universidade Federal da Paraíba, Programa de Pós-Graduação em Enfermagem, João Pessoa, PB, Brasil.

**Keywords:** Conhecimentos, Atitudes e Prática em Saúde, Automedicação, Estudantes, Universidades

## Abstract

**Objetivo:**

Avaliar o conhecimento, atitudes e práticas de automedicação e fatores associados a este comportamento entre estudantes da área da saúde.

**Método:**

Estudo transversal conduzido em uma universidade pública de Minas Gerais com 237 estudantes. A coleta de dados ocorreu via formulário estruturado para obtenção de variáveis sociodemográficas e sobre conhecimento, atitudes e práticas da automedicação. Realizou-se análise descritiva e modelo de regressão.

**Resultados:**

A maioria (84,8%) referiu praticar a automedicação e demonstrou alta favorabilidade para o comportamento; 57,7% demonstraram conhecimento adequado. Estudantes dos anos iniciais apresentaram maiores chances de conhecimento inadequado. Atitudes favoráveis associaram-se significativamente à faixa etária de 18 a 20 anos e a baixa renda. A maior prática de automedicação vinculou-se estatisticamente a jovens e autodeclarados pretos.

**Conclusão:**

A elevada ocorrência da automedicação e a falsa percepção de autonomia técnica evidenciam a medicalização da vida como resposta às pressões acadêmicas. Os resultados demonstraram vulnerabilidades associadas às desigualdades estruturais e reforçam a necessidade de intervenções institucionais voltadas à segurança medicamentosa.

## INTRODUÇÃO

No contexto da educação em saúde, o modelo denominado Conhecimento, Atitude e Prática (CAP) representa uma ferramenta essencial. Esta abordagem sistêmica que permite avaliar o que as pessoas sabem, como pensam e como agem diante de um determinado problema ou prática em saúde é fundamental para identificar riscos comportamentais específicos^(1)^. O conhecimento orienta as atitudes, que por sua vez, motivam as práticas, as quais, se consistentes, reforçam atitudes e ampliam determinado conhecimento^(1)^. Inquéritos do tipo CAP são amplamente utilizados para elucidar comportamentos em saúde, incluindo a automedicação em estudantes universitários^(2,3,4,5,6,7,8,9,10)^. Nesse grupo específico, o consumo de medicamentos sem prescrição de profissional habilitado é uma prática recorrente e de alta prevalência, especialmente entre estudantes da área da saúde^(2,3,4,5,6,7,8,9,10,11,12)^.

Entre universitários, as motivações para a automedicação fundamentam-se na busca pela otimização do tempo e na conveniência frente às pressões do cotidiano acadêmico. O alívio imediato de sintomas comuns, como cefaleias, quadros gripais e dismenorreia, constitui a principal justificativa para o uso de fármacos sem orientação profissional^(4,5,6,7,8,9,10,11,12)^. Somado a isso, as lacunas no sistema de saúde, manifestadas na morosidade do atendimento e na dificuldade de agendamento de consultas, atuam como fatores determinantes que impelem os jovens a essa prática. No entanto, tal cenário favorece o consumo indiscriminado de medicamentos, elevando os riscos de eventos adversos e configurando um grave desafio à segurança do paciente e ao uso racional^(2,4,7,13)^. A automedicação, além de comprometer o uso seguro de medicamentos, gera custos desnecessários ao sistema de saúde derivados do atraso no diagnóstico de doenças, interações medicamentosas, reações adversas graves e resistência antimicrobiana^(10,13)^.

Pesquisas baseadas no modelo CAP demonstram que fatores como nível educacional, renda, gênero e contexto cultural são determinantes na prática da automedicação^(5,10)^. No Brasil, contudo, estudos que aplicam essa tríade a universitários da área da saúde ainda são incipientes^(3)^, especialmente no que tange à geração Z. Indivíduos pertencentes à geração Z, caracterizados pela hiperconectividade e por uma acentuada busca por autonomia, apresentam um perfil de vulnerabilidade para decisões de saúde temerárias^(14)^. O acesso irrestrito às informações digitais e o imediatismo na resolução de problemas, quando associados às pressões acadêmicas e tensões cotidianas, potencializam o consumo irracional de medicamentos isentos de prescrição médica^(15)^. Em estudantes da área da saúde, esses traços comportamentais convergem sinergicamente com o acesso facilitado a literaturas especializadas e o convívio com pares do segmento assistencial^(4,5,6,7,8,9,10,11)^.

No âmbito acadêmico, embora os estudantes apresentem maior acesso aos medicamentos, sobretudo na prática da automedicação, e acreditem deter domínio técnico, o limitado treinamento terapêutico e a subestimação dos riscos clínicos, muitas vezes mascarada por uma falsa percepção de autonomia técnica decorrente do contato precoce com a assistência, podem induzir a comportamentos negligentes^(4)^. Essa visão equivocada culmina em práticas inseguras e na ocorrência de reações adversas^(2,9)^. Nesse cenário, a busca pelo alívio imediato de sintomas configura um comportamento de risco que deve ser confrontado por uma formação acadêmica ativa^(4,5,6,7,8,9,10,11,12)^, voltada à segurança dos usuários e à mitigação do consumo indiscriminado de medicamentos. Diante desse panorama, a interseção entre CAP e automedicação de universitários da geração Z emerge como um campo crítico de estudo e um desafio às instituições de ensino.

A universidade representa um espaço estratégico para o desenvolvimento de ações que desestimulem a automedicação indiscriminada e promovam o uso racional de medicamentos, a fim de capacitar o futuro profissional a reconhecer os limites do autocuidado e a importância da monitorização clínica. Diante disso, este estudo objetivou avaliar o conhecimento, atitudes e práticas de automedicação e fatores associados a este comportamento entre estudantes da área da saúde de uma universidade pública em Minas Gerais, com vistas a subsidiar intervenções educativas e políticas institucionais que mitiguem os riscos associados à automedicação e fortaleçam a cultura de segurança e o uso seguro de medicamentos.

## MÉTODO

### Desenho do Estudo

Trata-se de um estudo transversal.

### Local

Estudo realizado em uma universidade pública federal localizada na região Centro-Oeste de Minas Gerais.

### Critérios de Seleção

A população do estudo foi composta por estudantes dos cursos de Enfermagem, Medicina, Farmácia e Bioquímica. Os critérios de inclusão foram: idade igual ou superior a 18 anos e matrícula regular na instituição de ensino. Foram excluídos os estudantes sem endereço eletrônico ativo ou que apresentaram falhas técnicas no recebimento do instrumento de pesquisa via *e-mail*.

### Definição da Amostra

O cálculo amostral baseou-se em uma população de 692 estudantes, considerando-se a prevalência de automedicação de 63,9%, identificada em estudo com características e objetivos similares ao presente trabalho^(4)^. Adotou-se margem de erro de 5% e nível de confiança de 95%, resultando em uma amostra mínima estimada de 237 participantes. Para garantir a representatividade, a amostra foi estratificada de forma proporcional por curso e período acadêmico.

### Coleta de Dados

A coleta foi realizada *online*, entre fevereiro e agosto de 2024, por meio de formulário do *Google Forms*, enviado via *e-mail* institucional com convite e *link* de acesso. Após o aceite no Termo de Consentimento Livre e Esclarecido (TCLE), os participantes responderam a um questionário validado^(3)^ intitulado “Conhecimento, atitude e práticas da automedicação em estudantes universitários”, composto por 38 itens divididos em três seções:

*Seção A* – Caracterização sociodemográfica, acadêmica e de saúde: composta por questões direcionadas à identificação da idade, sexo, raça/cor, renda familiar, moradia atual, natureza da instituição de ensino, curso e período, acesso ao serviço de saúde e avaliação do estado de saúde.

*Seção B* – Conhecimento e prática sobre a automedicação: obtido de forma indireta a partir das respostas sobre a prática da automedicação. Para avaliar o conhecimento foram consideradas como referência as informações do banco de dados Drugs.com (acesso aberto)^(16)^. O conhecimento foi considerado adequado se as informações relatadas pelos participantes foram consideradas corretas pelo banco de dados além de não assinalar nenhum campo de dúvidas sobre a indicação, contraindicação, dosagem/administração, duração máxima de uso, potenciais reações adversas, interações medicamentosas, precauções ou outras dúvidas.

*Seção C* – Crenças e atitudes sobre a automedicação: foi mensurada a partir de uma escala tipo *Likert* de cinco pontos que varia de um (discordo totalmente) até cinco (concordo totalmente). A soma das respostas dividida pelo número de itens gera uma pontuação que pode variar entre 1 e 5; quanto maior o escore, mais favorável o estudante está para praticar a automedicação.

A prática da automedicação foi analisada com base no relato de uso de medicamentos nos últimos 15 dias, incluindo sintomas, tipo e uso dos medicamentos (nome, dose, via, posologia, efeitos percebidos e justificativas).

### Análise e Tratamento dos Dados

A análise estatística foi realizada no *software Statistical Package for the Social Sciences* (SPSS), versão 21.0. Utilizou-se estatística descritiva, com frequências absoluta e relativa para variáveis categóricas, e médias com desvio padrão (DP) ou medianas com intervalos interquartílicos (IQ), conforme a distribuição dos dados. A normalidade foi verificada pelo teste de *Kolmogorov-Smirnov*. Para as associações, empregou-se o teste qui-quadrado de *Pearson*. As variáveis dependentes foram: prática de automedicação (dicotômica), conhecimento (adequado/inadequado) e atitude (≤ 3 ou >3). As variáveis independentes abrangeram os blocos demográfico, socioeconômico, acadêmico e de uso de medicamentos. Variáveis com p < 0,20 na análise bivariada foram inseridas no modelo de regressão logística para cálculo da *Odds Ratio* (OR). Adotou-se o nível de significância de p < 0,05.

### Aspectos Éticos

O estudo foi aprovado pelo Comitê de Ética em Pesquisa da instituição (Parecer nº 4.055.445, CAAE 30696620.0.0000.5545) e seguiu todas as recomendações da Resolução nº 466/2012 do Conselho Nacional de Saúde (CNS). Todos os participantes assinaram o TCLE digitalmente, com garantia de anonimato e uso exclusivo dos dados para fins científicos.

## RESULTADOS

Dentre os 252 questionários recebidos, 15 foram excluídos por terem sido preenchidos de modo incompleto. Portanto, a amostra foi constituída de 237 respondentes. A Tabela 1 ilustra o total de estudantes avaliados e, cujas diferenças estatisticamente significantes ocorreram nas variáveis: ano do curso (p < 0,001) em relação ao conhecimento; faixa etária (p = 0,046) e renda (p < 0,010) em relação à atitude; e, faixa etária (p = 0,028), sexo (p = 0,048) e cor de pele (p = 0,001) em relação à prática de automedicação.

**Tabela 1:** Características sociodemográficas, acadêmicas e de saúde em relação ao conhecimento, às atitudes e às práticas dos estudantes universitários – Divinópolis, MG, Brasil, 2024.

Características dos estudantes	Conhecimento (n = 201)				Atitudes (n = 237)				Práticas (n = 237)		
	Adequado n (%)	Inadequado n (%)	p**		≤ 3,0 n (%)	> 3,0 n (%)	p**		Sim n (%)	Não n (%)	p**
Faixa etária											
≥ 18 e ≤ 20 anos	22 (10,9)	28 (13,9)	0,066		11 (4,6)	54 (22,8)	**0,046**		50 (21,1)	15 (6,3)	**0,028**
> 20 e ≤ 23 anos	50 (24,9)	33 (16,4)			32 (13,5)	67 (28,3)			83 (35,0)	16 (6,8)	
> 23 e ≤ 25 anos	44 (21,9)	24 (11,9)			25 (10,5)	48 (20,3)			68 (28,7)	5 (2,1)	
Sexo											
Feminino	100 (49,8)	76 (37,8)	0,324		56 (23,6)	147 (62,0)	0,234		176 (74,3)	27 (11,4)	**0,048**
Masculino	16 (8,0)	09 (4,5)			12 (5,1)	22 (9,3)			25 (10,5)	9 (3,8)	
Curso de graduação											
Enfermagem	32 (15,9)	25 (12,4)	0,822		26 (11,0)	46 (19,4)	0,225		57 (24,1)	15 (6,3)	0,232
Medicina	25 (12,4)	15 (7,5)			10 (4,2)	33 (13,9)			40 (16,9)	3 (1,3)	
Farmácia	34 (16,9)	23 (11,4)			22 (9,3)	46 (19,4)			57 (24,1)	11 (4,6)	
Bioquímica	25 (2,4)	22 (10,9)			10 (4,2)	44 (18,6)			47 (19,8)	7 (3,0)	
Ano de curso											
Primeiro e Segundo ano	38 (18,9)	60 (29,9)	**0,001**		32 (13,5)	90 (38,0)	0,688		98 (41,4)	24 (10,1)	0,245
Terceiro e Quarto ano	59 (29,4)	22 (10,9)			29 (12,2)	64 (27,0)			81 (34,2)	12 (5,1)	
Quinto e Sexto ano	19 (9,5)	03 (1,5)			07 (3,0)	15 (6,3)			22 (9,3)	0 (0,0)	
Renda familiar mensal*											
≤ 01 SM	24 (11,9)	12 (6,0)	0,675		08 (3,4)	37 (15,6)	**0,010**		36 (15,2)	9 (3,8)	0,497
> 01 SM e ≤ 02	23 (11,4)	19 (9,5)			14 (5,9)	35 (14,8)			42 (17,7)	7 (3,0)	
> 02 SM e ≤ 03	24 (11,9)	20 (10,0)			13 (5,5)	41 (17,3)			44 (18,6)	10 (4,2)	
> 03 SM	45 (22,4)	34 (16,9)			33 (13,9)	56 (23,6)			79 (33,3)	10 (4,2)	
Raça/Cor											
Branca	72 (35,8)	53 (26,4)	0,865		41 (17,3)	103 (43,5)	0,885		125 (52,7)	19 (8,0)	**0,001**
Parda	34 (16,9)	23 (11,4)			17 (7,2)	45 (19,0)			57 (24,1)	05 (2,1)	
Preta	10 (5,0)	09 (4,5)			10 (4,2)	21 (8,9)			19 (8,0)	12 (5,1)	
Acesso ao serviço de Saúde											
Público exclusivo	60 (29,9)	44 (21,9)	0,415		31 (13,1)	90 (38,0)	0,296		104 (43,9)	17 (7,2)	0,613
Público/Plano	46 (22,9)	29 (14,4)			42 (17,7)	46 (19,4)			75 (31,6)	13 (5,5)	
Plano/Particular	10 (5,0)	12 (6,0)			05 (2,1)	23 (9,7)			22 (9,3)	6 (2,5)	
Classificação do estado de saúde											
Muito Bom	26 (12,9)	15 (7,5)	0,098		12 (5,1)	38 (16,0)	0,241		41 (17,3)	9 (3,8)	0,617
Bom	59 (29,4)	35 (17,4)			38 (16,0)	74 (31,2)			94 (39,7)	18 (7,6)	
Ruim	31 (15,4)	35 (17,4)			18 (7,6)	57 (24,1)			66 (27,8)	9 (3,8)	

*Salário mínimo em 2024: R$1412,00;

**Teste Qui-Quadrado.

A maioria dos entrevistados (84,8%) relatou o comportamento de automedicação, e mais da metade (57,7%) referiu essa prática três ou mais vezes, nos últimos 15 dias. Em relação ao consumo, quase totalidade da amostra (96,5%) usou medicamentos de venda livre, com destaque para os analgésicos (70,1%) e sem relato de reações adversas (88,5%). Os principais motivos de saúde incluíram cefaleia (88,6%) e doenças do aparelho respiratório (67,2%). Pouco mais da metade (57,7%) da amostra apresentou conhecimento adequado sobre os medicamentos utilizados e a maioria (76,6%) afirmou que o conhecimento pessoal foi a principal fonte de informação para a prática de automedicação (Tabela 2).

**Tabela 2:** Práticas e conhecimento de estudantes universitários frente à automedicação – Divinópolis, MG, Brasil, 2024.

Conhecimento e práticas da automedicação	n (%)
Prática da automedicação	201 (84,8)
Motivo da automedicação*	
Dor de cabeça	178 (88,6)
Problemas em vias aéreas superiores	135 (67,2)
Cólica menstrual	84 (41,8)
Dor osteomuscular	76 (37,8)
Problemas gastrintestinais	72 (35,8)
Problemas emocionais	65 (32,3)
Contracepção	56 (27,9)
Problemas do sono	52 (25,9)
Febre	52 (25,9)
Problemas de pele	50 (24,9)
Justificativa para utilização*	
Decisão própria por conhecimento prévio	154 (76,6)
Repetição por decisão própria devido prescrição anterior	85 (42,3)
Indicação de Farmacêutico	30 (14,9)
Indicação de familiares	24 (11,9)
Indicação de amigos	16 (8,0)
Indicação de Balconista	7 (3,5)
Outros	4 (2,0)
Conhecimento*	
Adequado	116 (57,7)
Inadequado	85 (42,3)
Causas de conhecimento inadequado (n = 85)	
Dúvidas sobre tempo de utilização	42 (49,4)
Dúvidas sobre reações adversas	34 (40,0)
Dúvidas sobre dosagem	30 (35,3)
Dúvidas sobre interações medicamentosas	27 (31,8)
Dúvidas sobre contraindicação	25 (29,4)
Dúvidas sobre precaução durante o uso	21 (24,7)
Dúvidas sobre indicação	6 (7,0)
Erro de dosagem durante o uso	5 (5,9)

*Nas variáveis 'Motivo da automedicação', 'Justificativa para utilização' e ‘Conhecimento’, as porcentagens foram calculadas sobre o total de estudantes que referiram a prática de automedicação (n = 201). O somatório dos números absolutos excede o total de participantes devido à possibilidade de múltiplas respostas.

A Tabela 3 apresenta as respostas acerca das atitudes em relação à automedicação. A mediana das atitudes em relação à automedicação foi de 3,41 (QT25 2,932–QT75 3,864). As questões que apresentaram maior pontuação de atitude, ou seja, maior favorabilidade para a realização do comportamento, envolveram a influência da facilidade de comprar medicamentos de venda livre (77,6%), a utilização de medicamentos para sanar problemas comuns (75,9%), a prática da automedicação para obtenção de alívio rápido e pela conveniência (75,5%) e o tempo de espera para consulta com um profissional da saúde (70,0%).

**Tabela 3:** Distribuição de respostas aos itens do questionário de atitudes de estudantes universitários sobre a automedicação – Divinópolis, MG, Brasil, 2024.

Atitudes para automedicação	Discordo totalmente n (%)	Discordo n (%)	Não sei n (%)	Concordo n (%)	Concordo Totalmente n (%)
1. Dificuldades para acessar o sistema de saúde e a decisão da automedicação	40 (16,9)	26 (11,0)	55 (23,2)	52 (21,9)	64 (27,0)
2. Utilização de medicamentos diante problemas comuns	15 (6,3)	22 (9,3)	20 (8,4)	53 (22,4)	127 (53,6)
3. Utilização de medicamentos diante emergências	20 (8,4)	26 (11,0)	35 (14,8)	67 (28,3)	89 (37,6)
4. Utilização de medicamentos indicados por familiares ou amigos	54 (22,8)	51 (21,5)	65 (27,4)	36 (15,2)	31 (13,1)
5. Tempo de espera para consulta com um profissional da saúde e a decisão para automedicação	23 (9,7)	22 (9,3)	26 (11,0)	49 (20,7)	117 (49,4)
6. Falta de confiança em orientações de um profissional da saúde e a decisão para a automedicação	73 (30,8)	45 (19,0)	33 (13,9)	41 (17,3)	45 (19,0)
7. Reutilização de receita antiga e a facilidade para a automedicação	49 (20,7)	32 (13,5)	44 (18,6)	52 (21,9)	60 (25,3)
8. Ter estoque de medicamentos	31 (13,1)	32 (13,5)	31 (13,1)	43 (18,1)	100 (42,2)
9. Influência de propagandas de medicamentos e a decisão para automedicação	131 (55,3)	29 (12,2)	27 (11,4)	26 (11,0)	24 (10,1)
10. Busca de informações para a automedicação	29 (12,2)	22 (9,3)	40 (16,9)	52 (21,9)	94 (39,7)
11. Falta de tempo para procura de um profissional da saúde e a decisão da automedicação	21 (8,9)	37 (15,6)	31 (13,1)	58 (24,5)	90 (38,0)
12. Influência de medicamentos utilizados anteriormente e a decisão da automedicação	14 (5,9)	25 (10,5)	49 (20,7)	61 (25,7)	88 (37,1)
13. Influência de familiares sobre a automedicação	46 (19,4)	23 (9,7)	43 (18,1)	55 (23,2)	70 (29,5)
14. A prática da automedicação para reduzir faltas às aulas ou no trabalho	46 (19,4)	18 (7,6)	45 (19,0)	42 (17,7)	86 (36,3)
15. Relação da automedicação com a manutenção de um papel ativo nos cuidados à saúde	55 (23,2)	42 (17,7)	64 (27,0)	49 (20,7)	27 (11,4)
16. Relação de reduzir custos com consultas e exames e a automedicação	44 (18,6)	48 (20,3)	51 (21,5)	38 (16,0)	56 (23,6)
17. Decisão para a prática da automedicação envolvendo problemas de saúde menores	56 (23,6)	38 (16,0)	43 (18,1)	51 (21,5)	49 (20,7)
18. Segurança para a prática da automedicação	18 (7,6)	49 (20,7)	77 (32,5)	61 (25,7)	32 (13,5)
19. Influência da facilidade de comprar medicamentos de venda livre e a prática da automedicação	12 (5,1)	14 (5,9)	27 (11,4)	54 (22,8)	130 (54,9)
20. Prática da automedicação para obtenção de alívio rápido e por ser conveniente	12 (5,1)	21 (8,9)	25 (10,5)	68 (28,7)	111 (46,8)
21. Concordância de que medicamentos de venda livre possam ser acessíveis para automedicação	52 (21,9)	41 (17,3)	25 (10,5)	47 (19,8)	73 (30,4)
22. Amizade com profissionais ou estudante da saúde e a influência para à automedicação	33 (13,9)	30 (12,7)	30 (12,7)	59 (24,9)	85 (35,9)

Na análise da regressão logística, identificou-se que estudantes dos primeiros anos dos cursos (OR: 10,00; IC 95%: 2,77–35,10) apresentaram maiores chances de ter conhecimento inadequado; aqueles que classificaram o estado de saúde como bom (OR: 0,525; IC 95%: 0,27–0,99) apresentaram menores chances de ter conhecimento inadequado. A chance de estudantes com idade entre 18 e 20 anos (OR: 1,35; IC 95%: 1,32–7,75) e que possuíam renda familiar ≤ um salário mínimo (OR: 2,72; IC 95%: 1,13–6,55) foi significantemente maior de terem uma atitude mais favorável para o comportamento da automedicação, quando comparados a outras idades e rendas. As variáveis idade entre 18 e 20 anos (OR: 4,46; IC 95%: 1,43–13,90) e cor/raça preta (OR: 4,15; IC 95%: 1,74–9,91) mostraram-se associadas a uma maior chance para a prática da automedicação; em contrapartida, ser do sexo masculino esteve relacionado a uma chance menor para essa prática (OR: 0,36; IC 95%: 0,14–0,94).

## DISCUSSÃO

Os resultados deste estudo evidenciaram elevada prática de automedicação entre estudantes universitários da área da saúde. A maioria dos participantes relatou automedicação, com uma frequência superior a três vezes em mais da metade dos casos (57,7%). Esse comportamento foi motivado, prioritariamente, pela busca por soluções imediatas para alívio de sintomas relacionados à cefaleia e consumo de analgésicos.

Quanto ao conhecimento, embora mais da metade dos estudantes (57,7%) tenha apresentado compreensão adequada sobre automedicação, este índice é inferior aos estudos prévios e insuficiente para garantir a segurança medicamentosa^(4,7,10)^. O desconhecimento dos riscos entre estudantes ingressantes reforça que a inserção de conteúdos sobre farmacologia e uso racional de medicamentos nos currículos da área da saúde não deve visar uma “automedicação consciente”, mas sim capacitar o estudante a reconhecer a necessidade de auxílio profissional e a combater o uso irracional^(13,17)^. O ensino deve focar na prevenção de danos e na desconstrução da ideia de que o saber técnico autoriza a autoprescrição.

As atitudes favoráveis à automedicação refletem traços da geração Z, como a busca por autonomia e praticidade. No entanto, essa autonomia manifesta-se como uma vulnerabilidade digital, onde a facilidade de acesso a informações e medicamentos de venda livre suplanta a cautela clínica. Esse imediatismo geracional, aliado ao comportamento digital, especialmente de recorrer frequentemente à *internet* e às redes sociais, mascara os riscos inerentes a prática^(15,18)^. Esse déficit de percepção de risco, exige que as instituições de ensino problematizem o impacto da cultura do “autodiagnóstico *online*”.

A reutilização de prescrições antigas (42,3%) e a autoconfiança baseada em experiências prévias revelam uma falha na percepção de segurança. Barreiras no acesso aos serviços de saúde, tais como longos tempos de espera, escassez de profissionais e custos elevados, embora reais e identificadas por outros autores^(9,13,18)^, não justificam a automedicação; antes, configuram problemas estruturais que podem levar os indivíduos à adoção de práticas de risco, com potencial comprometimento da segurança do usuário em longo prazo. Fatores socioeconômicos e raciais influenciaram o comportamento de automedicação, com maior prevalência entre estudantes de menor renda familiar e autodeclarados pretos. Esses achados sugerem que a automedicação pode ser um reflexo das desigualdades de acesso^(6)^, mas também sinalizam a medicalização da vida como uma resposta paliativa a iniquidades sistêmicas e sociais, incluindo experiências de discriminação, desconfiança no sistema de saúde e influência de saberes populares^(19)^.

O expressivo consumo de analgésicos, que foram suporte para o alívio de cefaleia, queixa prevalente e consonante com outros autores^(10,12)^, sugere uma potencial interseção crítica entre automedicação e o fenômeno da medicalização da vida no contexto universitário. Esse ambiente, muitas vezes, empurra o estudante para uma lógica de performance que resulta em sofrimento psíquico, cuja tendência de tratamento é apenas medicamentoso^(20)^. Deste modo, é plausível inferir que, sob elevados níveis de estresse e ansiedade, estudantes da geração Z recorram aos analgésicos para o alívio imediato de manifestações somáticas (como cefaleias tensionais) decorrentes de pressões emocionais^(21)^. Assim, a utilização de analgésicos para mitigar o mal-estar psíquico transvestido de dor física configura um comportamento de risco que mascara o desgaste emocional e reforça a busca por soluções farmacológicas imediatas, em detrimento de estratégias de cuidado integral e do uso racional de medicamentos.

A dependência da *internet* como fonte de informação (*YouTube*, *Instagram*, *TikTok*) potencializa a disseminação de informações sem embasamento científico^(22,23)^. O fato de o conhecimento técnico isolado não prevenir a prática, achado evidenciado entre a maioria dos participantes nos diferentes cursos, indica que a formação acadêmica (nos diferentes níveis) precisa evoluir de uma transmissão passiva de informações para uma construção de atitudes críticas que desestimulem o uso de medicamentos sem supervisão profissional. A compreensão desse comportamento é essencial para intervenções eficazes.

Entretanto, o que muitas vezes é rotulado como “autonomia” da geração Z, na verdade, reforça a medicalização da vida e o uso indiscriminado, em detrimento de uma visão coletiva e segura de saúde^(21)^. Essa postura individualista é preocupante na formação em saúde, pois pode comprometer a futura atuação ética e a capacidade de oferecer um cuidado humano e tecnicamente responsável. Portanto, as instituições de ensino devem investir em campanhas educativas a fim de combater a automedicação e promover o uso racional de medicamentos. Essas ações devem utilizar estratégias digitais para oferecer alternativas reais de cuidado, como o incentivo à telemedicina e o aconselhamento com profissionais habilitados, desassociando a prática da automedicação do conceito de autocuidado^(8,21,23)^. O engajamento em comunidades *online* e histórias reais podem auxiliar na mudança de comportamento, focando na segurança e na responsabilidade clínica^(24)^.

Apesar das limitações de viés de memória decorrente da coleta autorrelatada e a abrangência restrita da amostra, os achados são cruciais por focalizarem a nova geração de profissionais da saúde. Torna-se imprescindível que os currículos dos cursos da área da saúde incorporem a prática profissional crítica, centrada na mitigação do consumo de medicamentos e no fortalecimento de políticas de regulação. A adoção do modelo CAP deve, portanto, servir para embasar estratégias multidisciplinares que protejam o estudante e o sistema de saúde do uso irracional de medicamentos.

## CONCLUSÃO

A lacuna no conhecimento técnico identificada em parcela significativa dos estudantes evidencia a vulnerabilidade desse grupo a riscos sanitários e reforça o fenômeno da medicalização da vida como resposta a demandas acadêmicas e sociais. Ademais, as disparidades observadas entre estudantes de menor renda e autodeclarados pretos ressaltam o impacto das desigualdades estruturais no acesso à informação qualificada em saúde. Conclui-se pela necessidade imperativa de implementar estratégias educacionais no ensino superior que transcendam a teoria farmacológica, focando no combate ao uso irracional de fármacos e na promoção da segurança medicamentosa, visando a formação de profissionais críticos e comprometidos com uma assistência segura, ética e equitativa.

## Data Availability

Todo o conjunto de dados que dá suporte aos resultados deste estudo foi publicado no próprio artigo.

# Translation: Conocimientos, actitudes y prácticas sobre la automedicación entre estudiantes universitarios de la Generación Z*

**DOI:** 10.1590/1980-220X-REEUSP-2025-0573es

**Authors:** Hemelly Nogueira Guimarães Silveira, Silvia Regina Secoli, Aline Carrilho Menezes, Mailson Marques de Sousa, Stéfanie de Souza Rocha Ferreira, Flávia de Oliveira, Silmara Nunes Andrade, Danilo Donizetti Trevisan

**Affiliations:** 1 Universidade Federal de São João del Rei, Programa de Pós-Graduação em Enfermagem, Divinópolis, MG, Brasil.; 2 Universidade de São Paulo, Escola de Enfermagem, Programa de Pós-Graduação em Enfermagem na Saúde do Adulto, São Paulo, SP, Brasil.; 3 Universidade Federal de São João del Rei, Departamento de Enfermagem, Divinópolis, MG, Brasil.; 4 Universidade Federal da Paraíba, Programa de Pós-Graduação em Enfermagem, João Pessoa, PB, Brasil.

**Keywords:** Conocimientos, Actitudes y Práctica en Salud, Automedicación, Estudiantes, Universidades

## Abstract

**Objetivo:**

Evaluar los conocimientos, las actitudes y las prácticas de automedicación, así como los factores asociados a este comportamiento, entre los estudiantes del área de la salud.

**Método:**

Estudio transversal realizado en una universidad pública de Minas Gerais con 237 estudiantes. La recolección de datos se llevó a cabo mediante un formulario estructurado para obtener variables sociodemográficas y sobre conocimientos, actitudes y prácticas de automedicación. Se realizó un análisis descriptivo y un modelo de regresión.

**Resultados:**

La mayoría (84,8 %) afirmó practicar la automedicación y mostró una alta aceptación de este comportamiento; el 57,7 % demostró un conocimiento adecuado. Los estudiantes de los primeros años presentaron mayores probabilidades de tener un conocimiento inadecuado. Las actitudes favorables se asociaron significativamente con el grupo de edad de 18 a 20 años y con los bajos ingresos. La mayor práctica de automedicación se relacionó estadísticamente con los jóvenes y con quienes se autodefinieron como negros.

**Conclusión:**

La elevada incidencia de la automedicación y la falsa percepción de autonomía técnica evidencian la medicalización de la vida como respuesta a las presiones académicas. Los resultados demostraron vulnerabilidades asociadas a las desigualdades estructurales y refuerzan la necesidad de intervenciones institucionales orientadas a la seguridad farmacológica.

## INTRODUCCIÓN

En el contexto de la educación en salud, el modelo denominado Conocimientos, Actitudes y Prácticas (CAP) representa una herramienta esencial. Este enfoque sistémico, que permite evaluar lo que las personas saben, cómo piensan y cómo actúan ante un determinado problema o práctica en salud, es fundamental para identificar riesgos conductuales específicos^(1)^. El conocimiento orienta las actitudes, que a su vez motivan las prácticas; estas, si son consistentes, refuerzan las actitudes y amplían determinados conocimientos^(1)^. Las encuestas de tipo CAP se utilizan ampliamente para esclarecer comportamientos en materia de salud, incluida la automedicación en estudiantes universitarios^(2,3,4,5,6,7,8,9,10)^. En este grupo específico, el consumo de medicamentos sin receta de un profesional habilitado es una práctica recurrente y de alta prevalencia, especialmente entre los estudiantes del área de la salud^(2,3,4,5,6,7,8,9,10,11,12)^.

Entre los universitarios, las motivaciones para la automedicación se basan en la búsqueda de la optimización del tiempo y en la conveniencia frente a las presiones de la vida académica cotidiana. El alivio inmediato de síntomas comunes, como dolores de cabeza, cuadros gripales y dismenorrea, constituye la principal justificación para el uso de fármacos sin orientación profesional^(4,5,6,7,8,9,10,11,12)^. Además, las deficiencias del sistema de salud, que se manifiestan en la lentitud de la atención y en la dificultad para programar consultas, actúan como factores determinantes que empujan a los jóvenes a esta práctica. Sin embargo, este escenario favorece el consumo indiscriminado de medicamentos, elevando los riesgos de eventos adversos y configurando un grave desafío para la seguridad del paciente y el uso racional^(2,4,7,13)^. La automedicación, además de comprometer el uso seguro de los medicamentos, genera costos innecesarios para el sistema de salud derivados del retraso en el diagnóstico de enfermedades, interacciones medicamentosas, reacciones adversas graves y resistencia a los antimicrobianos^(10,13)^.

Las investigaciones basadas en el modelo CAP demuestran que factores como el nivel educativo, los ingresos, el género y el contexto cultural son determinantes en la práctica de la automedicación^(5,10)^. En Brasil, sin embargo, los estudios que aplican esta tríada a estudiantes universitarios del área de la salud aún son incipientes^(3)^, especialmente en lo que respecta a la generación Z. Los individuos pertenecientes a la generación Z, caracterizados por la hiperconectividad y por una marcada búsqueda de autonomía, presentan un perfil de vulnerabilidad ante decisiones de salud temerarias^(14)^. El acceso sin restricciones a la información digital y la inmediatez en la resolución de problemas, cuando se asocian con las presiones académicas y las tensiones cotidianas, potencian el consumo irracional de medicamentos de venta libre^(15)^. En los estudiantes del área de la salud, estos rasgos de comportamiento convergen sinérgicamente con el acceso facilitado a la literatura especializada y la convivencia con compañeros del sector asistencial^(4,5,6,7,8,9,10,11)^.

En el ámbito académico, aunque los estudiantes tienen mayor acceso a los medicamentos, sobre todo en la práctica de la automedicación, y creen tener dominio técnico, la formación terapéutica limitada y la subestimación de los riesgos clínicos, a menudo enmascarada por una falsa percepción de autonomía técnica derivada del contacto precoz con la atención sanitaria, pueden inducir a comportamientos negligentes^(4)^. Esta visión errónea culmina en prácticas inseguras y en la aparición de reacciones adversas^(2,9)^. En este escenario, la búsqueda del alivio inmediato de los síntomas constituye un comportamiento de riesgo que debe ser contrarrestado mediante una formación académica activa^(4,5,6,7,8,9,10,11,12)^, orientada a la seguridad de los usuarios y a la mitigación del consumo indiscriminado de medicamentos. Ante este panorama, la intersección entre la CAP y la automedicación de los universitarios de la generación Z surge como un campo crítico de estudio y un desafío para las instituciones educativas.

La universidad representa un espacio estratégico para el desarrollo de acciones que desalienten la automedicación indiscriminada y promuevan el uso racional de los medicamentos, con el fin de capacitar al futuro profesional para reconocer los límites del autocuidado y la importancia del seguimiento clínico. Ante esto, este estudio tuvo como objetivo evaluar los conocimientos, las actitudes y las prácticas de automedicación, así como los factores asociados a este comportamiento, entre los estudiantes del área de la salud de una universidad pública en Minas Gerais, con miras a respaldar intervenciones educativas y políticas institucionales que mitiguen los riesgos asociados a la automedicación y fortalezcan la cultura de seguridad y el uso seguro de los medicamentos.

## MÉTODO

### Diseño del Estudio

Se trata de un estudio transversal.

### Lugar

Estudio realizado en una universidad pública federal ubicada en la región centro-oeste de Minas Gerais.

### Criterios de Selección

La población del estudio estuvo compuesta por estudiantes de los cursos de Enfermería, Medicina, Farmacia y Bioquímica. Los criterios de inclusión fueron: edad igual o superior a 18 años y estar matriculado regularmente en la institución educativa. Se excluyó a los estudiantes sin dirección de correo electrónico activa o que presentaron fallas técnicas en la recepción del instrumento de investigación vía *correo electrónico*.

### Definiciónde la Muestra

El cálculo de la muestra se basó en una población de 692 estudiantes, considerando la prevalencia de automedicación del 63,9 %, identificada en un estudio con características y objetivos similares a los del presente trabajo^(4)^. Se adoptó un margen de error del 5 % y un nivel de confianza del 95 %, lo que dio como resultado una muestra mínima estimada de 237 participantes. Para garantizar la representatividad, la muestra se estratificó de manera proporcional por carrera y período académico.

### Recolección de Datos

La recolección se realizó en línea, entre febrero y agosto de 2024, mediante un formulario de Google Forms, enviado por correo electrónico institucional con una invitación y un enlacede acceso. Tras aceptar el Término de Consentimiento Libre e Informado, los participantes respondieron a un cuestionario validado^(3)^titulado “Conocimiento, actitud y prácticas de automedicación en estudiantes universitarios”, compuesto por 38 ítems divididos en tres secciones:

*Sección A* – Caracterización sociodemográfica, académica y de salud: compuesta por preguntas dirigidas a identificar la edad, el sexo, la raza/color, el ingreso familiar, la vivienda actual, el tipo de institución educativa, el curso y el período, el acceso a los servicios de salud y la evaluación del estado de salud.

*Sección B* – Conocimiento y práctica sobre la automedicación: obtenido de manera indirecta a partir de las respuestas sobre la práctica de la automedicación. Para evaluar el conocimiento se tomó como referencia la información de la base de datos Drugs.com (acceso abierto)^(16)^. El conocimiento se consideró adecuado si la información proporcionada por los participantes fue considerada correcta por la base de datos y no se señaló ningún campo de dudas sobre la indicación, contraindicación, dosificación/administración, duración máxima de uso, posibles reacciones adversas, interacciones medicamentosas, precauciones u otras dudas.

*Sección C* – Creencias y actitudes sobre la automedicación: se midió a partir de una escala tipo *Likert* de cinco puntos que varía de uno (totalmente en desacuerdo) a cinco (totalmente de acuerdo). La suma de las respuestas dividida por el número de ítems genera una puntuación que puede variar entre 1 y 5; cuanto mayor sea la puntuación, más favorable es la actitud del estudiante hacia la práctica de la automedicación.

La práctica de la automedicación se analizó basándose en el informe del uso de medicamentos en los últimos 15 días, incluyendo síntomas, tipo y uso de los medicamentos (nombre, dosis, vía, posología, efectos percibidos y justificaciones).

### Análisis y Tratamiento de los Datos

El análisis estadístico se realizó con el *software Statistical Package for the Social Sciences* (SPSS), versión 21.0. Se utilizó estadística descriptiva, con frecuencias absolutas y relativas para las variables categóricas, y medias con desviación estándar (DE) o medianas con intervalos intercuartílicos (IQ), según la distribución de los datos.

La normalidad se verificó mediante la prueba de *Kolmogorov-Smirnov*. Para las asociaciones, se empleó la prueba de Chi-cuadrado de *Pearson*. Las variables dependientes fueron: práctica de automedicación (dicotómica), conocimiento (adecuado/inadecuado) y actitud (≤3 o >3). Las variables independientes abarcaron los bloques demográfico, socioeconómico, académico y de uso de medicamentos. Las variables con p < 0,20 en el análisis bivariado se incluyeron en el modelo de regresión logística para el cálculo de la *Odds Ratio* (OR). Se adoptó un nivel de significación de p < 0,05.

### Aspectos Éticos

El estudio fue aprobado por el Comité de Ética en Investigación de la institución (Dictamen n.º 4.055.445, CAAE 30696620.0.0000.5545) y siguió todas las recomendaciones de la Resolución n.º 466/2012 del Consejo Nacional de Salud (CNS). Todos los participantes firmaron el término de consentimiento digitalmente, con garantía de anonimato y uso exclusivo de los datos con fines científicos.

## RESULTADOS

De los 252 cuestionarios recibidos, 15 fueron excluidos por estar incompletos. Por lo tanto, la muestra estuvo compuesta por 237 encuestados. La Tabla 1 ilustra el total de estudiantes evaluados, en los que se observaron diferencias estadísticamente significativas en las variables: año de carrera (p < 0,001) en relación con el conocimiento; grupo de edad (p = 0,046) e ingresos (p < 0,010) en relación con la actitud; y grupo de edad (p = 0,028), sexo (p = 0,048) y color de piel (p = 0,001) en relación con la práctica de la automedicación.

**Tabla 1:** Características sociodemográficas, académicas y de salud en relación con los conocimientos, las actitudes y las prácticas de los estudiantes universitários – Divinópolis, MG, Brasil, 2024.

Características de los estudiantes	Conocimientos (n = 201)				Actitudes (n = 237)				Prácticas (n = 237)		
	Adecuado n (%)	Inadecuado n (%)	p**		≤ 3,0 n (%)	> 3,0 n (%)	p**		Sí n (%)	No n (%)	p**
Grupo de edad											
≥ 18 y ≤ 20 años	22 (10,9)	28 (13,9)	0,066		11 (4,6)	54 (22,8)	**0,046**		50 (21,1)	15 (6,3)	**0,028**
> 20 y ≤ 23 años	50 (24,9)	33 (16,4)			32 (13,5)	67 (28,3)			83 (35,0)	16 (6,8)	
> 23 y ≤ 25 anõs	44 (21,9)	24 (11,9)			25 (10,5)	48 (20,3)			68 (28,7)	5 (2,1)	
Sexo											
Mujer	100 (49,8)	76 (37,8)	0,324		56 (23,6)	147 (62,0)	0,234		176 (74,3)	27 (11,4)	**0,048**
Hombres	16 (8,0)	09 (4,5)			12 (5,1)	22 (9,3)			25 (10,5)	9 (3,8)	
Grado											
Enfermería	32 (15,9)	25 (12,4)	0,822		26 (11,0)	46 (19,4)	0,225		57 (24,1)	15 (6,3)	0,232
Medicina	25 (12,4)	15 (7,5)			10 (4,2)	33 (13,9)			40 (16,9)	3 (1,3)	
Farmacia	34 (16,9)	23 (11,4)			22 (9,3)	46 (19,4)			57 (24,1)	11 (4,6)	
Bioquímica	25 (2,4)	22 (10,9)			10 (4,2)	44 (18,6)			47 (19,8)	7 (3,0)	
Curso											
Primer y segundo año	38 (18,9)	60 (29,9)	**0,001**		32 (13,5)	90 (38,0)	0,688		98 (41,4)	24 (10,1)	0,245
Tercer y cuarto año	59 (29,4)	22 (10,9)			29 (12,2)	64 (27,0)			81 (34,2)	12 (5,1)	
Quinto y sexto curso	19 (9,5)	03 (1,5)			07 (3,0)	15 (6,3)			22 (9,3)	0 (0,0)	
Renta familiar mensual*											
≤ 01 SM	24 (11,9)	12 (6,0)	0,675		08 (3,4)	37 (15,6)	**0,010**		36 (15,2)	9 (3,8)	0,497
> 01 SM y ≤ 02	23 (11,4)	19 (9,5)			14 (5,9)	35 (14,8)			42 (17,7)	7 (3,0)	
> 02 SM y ≤ 03	24 (11,9)	20 (10,0)			13 (5,5)	41 (17,3)			44 (18,6)	10 (4,2)	
> 03 SM	45 (22,4)	34 (16,9)			33 (13,9)	56 (23,6)			79 (33,3)	10 (4,2)	
Raza/Color											
Blanca	72 (35,8)	53 (26,4)	0,865		41 (17,3)	103 (43,5)	0,885		125 (52,7)	19 (8,0)	**0,001**
Parda	34 (16,9)	23 (11,4)			17 (7,2)	45 (19,0)			57 (24,1)	05 (2,1)	
Negra	10 (5,0)	09 (4,5)			10 (4,2)	21 (8,9)			19 (8,0)	12 (5,1)	
Acceso al servicio de Salud											
Público exclusivo	60 (29,9)	44 (21,9)	0,415		31 (13,1)	90 (38,0)	0,296		104 (43,9)	17 (7,2)	0,613
Público/Plano	46 (22,9)	29 (14,4)			42 (17,7)	46 (19,4)			75 (31,6)	13 (5,5)	
Público/Particular	10 (5,0)	12 (6,0)			05 (2,1)	23 (9,7)			22 (9,3)	6 (2,5)	
Clasificación del estado de salud											
Muy buena	26 (12,9)	15 (7,5)	0,098		12 (5,1)	38 (16,0)	0,241		41 (17,3)	9 (3,8)	0,617
Bueno	59 (29,4)	35 (17,4)			38 (16,0)	74 (31,2)			94 (39,7)	18 (7,6)	
Malo	31 (15,4)	35 (17,4)			18 (7,6)	57 (24,1)			66 (27,8)	9 (3,8)	

*Salario mínimo en 2024: 1412,00 R$;

**Prueba de chi-cuadrado.

La mayoría de los encuestados (84,8 %) declaró haber recurrido a la automedicación, y más de la mitad (57,7 %) señaló haberlo hecho tres o más veces en los últimos 15 días. En cuanto al consumo, casi la totalidad de la muestra (96,5 %) utilizó medicamentos de venta libre, destacando los analgésicos (70,1 %), sin que se informara de reacciones adversas (88,5 %). Los principales motivos de salud incluyeron la cefalea (88,6 %) y las enfermedades del aparato respiratorio (67,2 %). Poco más de la mitad (57,7 %) de la muestra presentó un conocimiento adecuado sobre los medicamentos utilizados y la mayoría (76,6 %) afirmó que el conocimiento personal fue la principal fuente de información para la práctica de la automedicación (Tabla 2).

**Tabla 2:** Prácticas y conocimientos de los estudiantes universitarios sobre la automedicación – Divinópolis, MG, Brasil, 2024.

Conocimientos y prácticas de automedicación	n (%)
Práctica de la automedicación	201 (84,8)
Motivo de la automedicación*	
Dolor de cabeza	178 (88,6)
Problemas en las vías respiratorias superiores	135 (67,2)
Dolor menstrual	84 (41,8)
Dolor musculoesquelético	76 (37,8)
Problemas gastrointestinales	72 (35,8)
Problemas emocionales	65 (32,3)
Anticoncepción	56 (27,9)
Problemas de sueño	52 (25,9)
Fiebre	52 (25,9)
Problemas de la piel	50 (24,9)
Justificación del uso*	
Decisión propia por conocimiento previo	154 (76,6)
Repetición por decisión propia debido a una prescripción anterior	85 (42,3)
Recomendación del farmacéutico	30 (14,9)
Recomendación de familiares	24 (11,9)
Recomendación de amigos	16 (8,0)
Recomendación de un dependiente	7 (3,5)
Otros	4 (2,0)
Conocimiento*	
Adecuado	116 (57,7)
Insuficiente	85 (42,3)
Causas del conocimiento inadecuado (n = 85)	
Dudas sobre el tiempo de uso	42 (49,4)
Dudas sobre las reacciones adversas	34 (40,0)
Dudas sobre la dosis	30 (35,3)
Dudas sobre interacciones farmacológicas	27 (31,8)
Dudas sobre contraindicaciones	25 (29,4)
Dudas sobre precauciones durante el uso	21 (24,7)
Dudas sobre la indicación	6 (7,0)
Error de dosificación durante el uso	5 (5,9)

*En las variables «Motivo de la automedicación», «Justificación del uso» y «Conocimiento», los porcentajes se calcularon sobre el total de estudiantes que indicaron practicar la automedicación (n = 201). La suma de los números absolutos supera el total de participantes debido a la posibilidad de respuestas múltiples.

La Tabla 3 presenta las respuestas sobre las actitudes hacia la automedicación. La mediana de las actitudes hacia la automedicación fue de 3,41 (QT25 2,932 - QT75 3,864). Las preguntas que obtuvieron una puntuación más alta en cuanto a la actitud, es decir, una mayor predisposición a realizar el comportamiento, se referían a la influencia de la facilidad para comprar medicamentos de venta libre (77,6 %), el uso de medicamentos para tratar problemas comunes (75,9 %), la práctica de la automedicación para obtener un alivio rápido y por comodidad (75,5 %) y el tiempo de espera para consultar a un profesional de la salud (70,0 %).

**Tabla 3:** Distribución de las respuestas a los ítems del cuestionario de actitudes de los estudiantes universitarios sobre la automedicación – Divinópolis, MG, Brasil, 2024.

Actitudes hacia la automedicación	Totalmente en desacuerdo n (%)	No estoy de acuerdo n (%)	No sé n (%)	Estoy de acuerdo n (%)	Totalmente de acuerdo n (%)
1. Dificultades para acceder al sistema sanitario y la decisión de automedicarse	40 (16,9)	26 (11,0)	55 (23,2)	52 (21,9)	64 (27,0)
2. Uso de medicamentos ante problemas comunes	15 (6,3)	22 (9,3)	20 (8,4)	53 (22,4)	127 (53,6)
3. Uso de medicamentos ante situaciones de emergencia	20 (8,4)	26 (11,0)	35 (14,8)	67 (28,3)	89 (37,6)
4. Uso de medicamentos recomendados por familiares o amigos	54 (22,8)	51 (21,5)	65 (27,4)	36 (15,2)	31 (13,1)
5. Tiempo de espera para consultar a un profesional sanitario y la decisión de automedicarse	23 (9,7)	22 (9,3)	26 (11,0)	49 (20,7)	117 (49,4)
6. Falta de confianza en las orientaciones de un profesional sanitario y la decisión de automedicarse	73 (30,8)	45 (19,0)	33 (13,9)	41 (17,3)	45 (19,0)
7. Reutilización de recetas antiguas y facilidad para la automedicación	49 (20,7)	32 (13,5)	44 (18,6)	52 (21,9)	60 (25,3)
8. Disponer de existencias de medicamentos	31 (13,1)	32 (13,5)	31 (13,1)	43 (18,1)	100 (42,2)
9. Influencia de la publicidad de medicamentos y la decisión de automedicarse	131 (55,3)	29 (12,2)	27 (11,4)	26 (11,0)	24 (10,1)
10. Búsqueda de información para la automedicación	29 (12,2)	22 (9,3)	40 (16,9)	52 (21,9)	94 (39,7)
11. Falta de tiempo para buscar un profesional sanitario y la decisión de automedicarse	21 (8,9)	37 (15,6)	31 (13,1)	58 (24,5)	90 (38,0)
12. Influencia de los medicamentos utilizados anteriormente y la decisión de automedicarse	14 (5,9)	25 (10,5)	49 (20,7)	61 (25,7)	88 (37,1)
13. Influencia de los familiares en la automedicación	46 (19,4)	23 (9,7)	43 (18,1)	55 (23,2)	70 (29,5)
14. La automedicación para reducir el absentismo escolar o laboral	46 (19,4)	18 (7,6)	45 (19,0)	42 (17,7)	86 (36,3)
15. Relación entre la automedicación y el mantenimiento de un papel activo en el cuidado de la salud	55 (23,2)	42 (17,7)	64 (27,0)	49 (20,7)	27 (11,4)
16. Relación entre la reducción de costes en consultas y pruebas y la automedicación	44 (18,6)	48 (20,3)	51 (21,5)	38 (16,0)	56 (23,6)
17. Decisión de automedicarse en caso de problemas de salud leves	56 (23,6)	38 (16,0)	43 (18,1)	51 (21,5)	49 (20,7)
18. Seguridad en la práctica de la automedicación	18 (7,6)	49 (20,7)	77 (32,5)	61 (25,7)	32 (13,5)
19. Influencia de la facilidad para adquirir medicamentos sin receta y la práctica de la automedicación	12 (5,1)	14 (5,9)	27 (11,4)	54 (22,8)	130 (54,9)
20. Práctica de la automedicación para obtener un alivio rápido y por ser conveniente	12 (5,1)	21 (8,9)	25 (10,5)	68 (28,7)	111 (46,8)
21. Acuerdo en que los medicamentos de venta libre puedan ser accesibles para la automedicación	52 (21,9)	41 (17,3)	25 (10,5)	47 (19,8)	73 (30,4)
22. Relación con profesionales o estudiantes de la salud y su influencia en la automedicación	33 (13,9)	30 (12,7)	30 (12,7)	59 (24,9)	85 (35,9)

En el análisis de regresión logística, se observó que los estudiantes de los primeros años de los cursos (OR: 10,00; IC del 95 %: 2,77–35,10) tenían más probabilidades de tener conocimientos inadecuados; mientras que aquellos que calificaron su estado de salud como bueno (OR: 0,525; IC 95 %: 0,27–0,99) presentaban menores probabilidades de tener conocimientos inadecuados. La probabilidad de que los estudiantes de entre 18 y 20 años (OR: 1,35; IC 95 %: 1,32–7,75) y que tenían un ingreso familiar ≤ un salario mínimo (OR: 2,72; IC 95 %: 1,13–6,55) de tener una actitud más favorable hacia el comportamiento de automedicación fue significativamente mayor, en comparación con otros grupos de edad e ingresos. Las variables edad entre 18 y 20 años (OR: 4,46; IC 95 %: 1,43–13,90) y raza negra (OR: 4,15; IC 95 %: 1,74–9,91) se asociaron con una mayor probabilidad de practicar la automedicación; por el contrario, ser de sexo masculino se relacionó con una menor probabilidad de esta práctica (OR: 0,36; IC 95 %: 0,14–0,94).

## DISCUSIÓN

Los resultados de este estudio pusieron de manifiesto una elevada práctica de automedicación entre los estudiantes universitarios del área de la salud. La mayoría de los participantes informó de automedicación, con una frecuencia superior a tres veces en más de la mitad de los casos (57,7 %). Este comportamiento se debió, principalmente, a la búsqueda de soluciones inmediatas para aliviar los síntomas relacionados con el dolor de cabeza y al consumo de analgésicos.

En cuanto al conocimiento, aunque más de la mitad de los estudiantes (57,7 %) demostró una comprensión adecuada sobre la automedicación, este índice es inferior al de estudios previos e insuficiente para garantizar la seguridad de los medicamentos^(4,7,10)^. El desconocimiento de los riesgos entre los estudiantes de primer año refuerza la idea de que la inclusión de contenidos sobre farmacología y uso racional de medicamentos en los planes de estudio del área de la salud no debe apuntar a una “automedicación consciente”, sino capacitar al estudiante para reconocer la necesidad de ayuda profesional y combatir el uso irracional^(13,17)^. La enseñanza debe centrarse en la prevención de daños y en desmontar la idea de que el conocimiento técnico autoriza la autoprescripción.

Las actitudes favorables a la automedicación reflejan rasgos de la generación Z, como la búsqueda de autonomía y practicidad. Sin embargo, esta autonomía se manifiesta como una vulnerabilidad digital, donde la facilidad de acceso a la información y a los medicamentos de venta libre suplanta la cautela clínica. Este inmediatismo generacional, aliado al comportamiento digital, especialmente al uso frecuente de *Internet* y las redes sociales, enmascara los riesgos inherentes a la práctica^(15,18)^. Este déficit de percepción del riesgo exige que las instituciones educativas analicen el impacto de la cultura del “autodiagnóstico en línea”.

La reutilización de recetas antiguas (42,3 %) y la autoconfianza basada en experiencias previas revelan una falla en la percepción de la seguridad. Las barreras en el acceso a los servicios de salud, tales como largos tiempos de espera, escasez de profesionales y costos elevados, aunque reales e identificadas por otros autores^(9,13,18)^, no justifican la automedicación; más bien, constituyen problemas estructurales que pueden llevar a las personas a adoptar prácticas de riesgo, con un potencial compromiso de la seguridad del usuario a largo plazo. Los factores socioeconómicos y raciales influyeron en el comportamiento de automedicación, con mayor prevalencia entre los estudiantes de menor ingreso familiar y los que se autodefinieron como negros. Estos hallazgos sugieren que la automedicación puede ser un reflejo de las desigualdades de acceso^(6)^, pero también señalan la medicalización de la vida como una respuesta paliativa a las iniquidades sistémicas y sociales, incluyendo experiencias de discriminación, desconfianza en el sistema de salud e influencia de los conocimientos populares^(19)^.

El consumo significativo de analgésicos, que sirvieron de apoyo para el alivio de la cefalea —una queja prevalente y en consonancia con otros autores^(10,12)^—, sugiere una posible intersección crítica entre la automedicación y el fenómeno de la medicalización de la vida en el contexto universitario.

Este entorno, a menudo, empuja al estudiante hacia una lógica de rendimiento que resulta en sufrimiento psíquico, cuya tendencia de tratamiento es únicamente farmacológica^(20)^. De este modo, es plausible inferir que, bajo elevados niveles de estrés y ansiedad, los estudiantes de la generación Z recurran a los analgésicos para el alivio inmediato de manifestaciones somáticas (como cefaleas tensionales) derivadas de presiones emocionales^(21)^. Por lo tanto, el uso de analgésicos para mitigar el malestar psíquico disfrazado de dolor físico constituye un comportamiento de riesgo que enmascara el desgaste emocional y refuerza la búsqueda de soluciones farmacológicas inmediatas, en detrimento de estrategias de atención integral y del uso racional de los medicamentos.

La dependencia de *Internet* como fuente de información (*YouTube*, *Instagram*, *TikTok*) potencia la difusión de información sin fundamento científico^(22,23)^. El hecho de que el conocimiento técnico aislado no prevenga esta práctica, hallazgo evidenciado entre la mayoría de los participantes en los diferentes cursos, indica que la formación académica (en los diferentes niveles) debe evolucionar de una transmisión pasiva de información a la construcción de actitudes críticas que desalienten el uso de medicamentos sin supervisión profesional. La comprensión de este comportamiento es esencial para intervenciones eficaces.

Sin embargo, lo que a menudo se etiqueta como “autonomía” de la generación Z, en realidad refuerza la medicalización de la vida y el uso indiscriminado, en detrimento de una visión colectiva y segura de la salud^(21)^. Esta postura individualista es preocupante en la formación en salud, ya que puede comprometer la futura actuación ética y la capacidad de ofrecer una atención humana y técnicamente responsable. Por lo tanto, las instituciones educativas deben invertir en campañas educativas para combatir la automedicación y promover el uso racional de los medicamentos. Estas acciones deben utilizar estrategias digitales para ofrecer alternativas reales de atención, como el fomento de la telemedicina y el asesoramiento con profesionales capacitados, desvinculando la práctica de la automedicación del concepto de autocuidado^(8,21,23)^. La participación en comunidades en línea y las historias reales pueden ayudar a cambiar el comportamiento, centrándose en la seguridad y la responsabilidad clínica^(24)^.

A pesar de las limitaciones del sesgo de memoria derivado de la Recolección de datos autoinformados y del alcance restringido de la muestra, los hallazgos son cruciales por centrarse en la nueva generación de profesionales de la salud. Es imprescindible que los planes de estudio de los cursos del área de la salud incorporen la práctica profesional crítica, centrada en la mitigación del consumo de medicamentos y en el fortalecimiento de las políticas de regulación. La adopción del modelo CAP debe, por lo tanto, servir para fundamentar estrategias multidisciplinarias que protejan al estudiante y al sistema de salud del uso irracional de medicamentos.

## CONCLUSIÓN

La brecha en el conocimiento técnico identificada en una parte significativa de los estudiantes evidencia la vulnerabilidad de este grupo ante los riesgos sanitarios y refuerza el fenómeno de la medicalización de la vida como respuesta a las demandas académicas y sociales. Además, las disparidades observadas entre los estudiantes de menores ingresos y los que se autodefinen como negros resaltan el impacto de las desigualdades estructurales en el acceso a información calificada en salud.

Se concluye que existe una necesidad imperiosa de implementar estrategias educativas en la educación superior que trasciendan la teoría farmacológica, centrándose en la lucha contra el uso irracional de fármacos y en la promoción de la seguridad de los medicamentos, con el objetivo de formar profesionales críticos y comprometidos con una atención segura, ética y equitativa.

## Data Availability

Todo el conjunto de datos que respalda los resultados de este estudio se publicó en el propio artículo.
